# RNA Aptamers for a tRNA-Binding Protein from *Aeropyrum pernix* with Homologous Counterparts Distributed Throughout Evolution

**DOI:** 10.3390/life10020011

**Published:** 2020-02-01

**Authors:** Senri Ohmori, Marina Wani, Saki Kitabatake, Yuka Nakatsugawa, Tadashi Ando, Takuya Umehara, Koji Tamura

**Affiliations:** 1 Department of Biological Science and Technology, Tokyo University of Science, 6-3-1 Niijuku, Katsushika-ku, Tokyo 125-8585, Japan; senri.omori@gmail.com (S.O.); m3_ks_5_1012@yahoo.co.jp (M.W.); 8310036@alumni.tus.ac.jp (S.K.); 8313068@alumni.tus.ac.jp (Y.N.); utumehara@mail.ecc.u-tokyo.ac.jp (T.U.); 2 Department of Applied Electronics, Tokyo University of Science, 6-3-1 Katsushika-ku, Tokyo 125-8585, Japan; tando@rs.tus.ac.jp; 3 Research Institute for Science and Technology, Tokyo University of Science, 2641 Yamazaki, Noda, Chiba 278-8510, Japan

**Keywords:** tRNA-binding protein (Trbp), *Aeropyrum pernix*, RNA aptamers, universal CCA, evolution

## Abstract

In the present *in vitro* selection study, we isolated and characterized RNA aptamers for a tRNA-binding protein (Trbp) from an extremophile archaeon *Aeropyrum pernix*. Trbp-like structures are frequently found not only in aminoacyl-tRNA synthetases but also in diverse types of proteins from different organisms. They likely arose early in evolution and have played important roles in evolution through interactions with key RNA structures. RNA aptamers specific for *A. pernix* Trbp were successfully selected from a pool of RNAs composed of 60 nucleotides, including a random 30-nucleotide region. From the secondary structures, we obtained a shortened sequence composed of 21 nucleotides, of which the 3′-terminal single stranded CA nucleotides are essential for binding. This may be related to the initial evolutionary role of the universal CCA-3′ terminus of tRNA in the interaction with Trbp-like structures.

## 1. Introduction

Trbp111, a tRNA-binding protein consisting of 111-amino acid polypeptides, was first identified in an extreme thermophile, *Aquifex aeolicus* [1]. According to the phylogenetic tree based on 16S rRNA proposed by Woese [2], this bacterium is considered to be the earliest branch of eubacteria. The Trbp111 protein is suggested to bind to the outer corner of the L-shaped tRNA as a homodimer [3,4], suggesting an evolutionary role in the formation of tRNAs by stabilizing RNA dimers. Trbp111-like proteins are evolutionarily conserved [1,5,6,7,8], and these structures are also involved in some aminoacyl-tRNA synthetases (aaRSs), ancient proteins that likely arose early in evolution [9]. For example, the tertiary structures of the C-terminal domains of methionyl-tRNA synthetase (MetRS-C) from archaeon species *Pyrococcus abyssi* [10] and *Nanoarchaeum equitans* [11] were found to resemble *A. aeolicus* Trbp111. They exist as homodimers, and each monomer possesses an oligonucleotide/oligosaccharide-binding fold (OB-fold).

Furthermore, we analyzed the binding of *N. equitans* MetRS-C to tRNA using a quartz crystal microbalance [11]. Interestingly, the binding of the split 3′-half tRNA species was stronger than that of the 5′-half species, suggesting that the interaction of the T-loop region of the 3′-half with a Trbp111-like structure may have initiated the formation of modern shaped tRNA [11].

**Figure 1:**
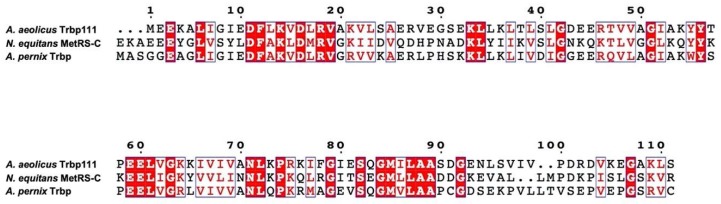
Multisequence alignment of *Aquifex aeolicus* Trbp111, *Nanoarchaeum equitans* MetRS-C, and *Aeropyrum pernix* Trbp (APE_0675.1). Similar amino acids are labeled with a white background and red font (white background), and conserved amino acids are labeled with a red background (white font).

However, the above MetRS-Cs were prepared as a part of gene-coded proteins, and an independent polypeptide sequence homologous to *A. aeolicus* Trbp111 has not been identified in *N. equitans*. *Aeropyrum pernix* is a species of extremophile archaeon in Crenarchaeota isolated from sediments in the sea off the coast of Japan [12]. Unlike *N. equitans*, an independent polypeptide homologous to *A. aeolicus* Trbp111 is coded in the genome of *A. pernix*, hereafter named “*A. pernix* Trbp”. In addition, multiple alignments created by Clustal Omega [13] show that *A. pernix* Trbp has high similarity to both *A. aeolicus* Trbp111 and *N. equitans* MetRS-C (Figure 1). 

Therefore, Trbp is likely an ancient protein involved in tRNA- and tRNA-related metabolism, and interactions with key RNA structures may have played a crucial role in evolution. *In vitro* selection of RNAs binding to Trbp to elucidate a potential interaction network has not yet been performed. Here, we report the isolation and characterization of the RNA aptamers for *A. pernix* Trbp. Based on the features of the secondary structures of the aptamers, binding analysis of the deleted and/or mutated aptamers bound to *A. pernix* Trbp was carried out using gel shift assays. 

## 2. Materials and Methods 

### 2.1. Plasmid Construction for Expression of A. pernix Trbp

The coding sequence for *A. pernix* Trbp (APE_0675.1) was amplified by PCR from genomic DNA (a gift from Dr. Yoshinori Koyama, National Institute of Advanced Industrial Science and Technology (AIST), Japan) using primers flanked with *Nde*I and *Bam*HI cut sites (synthesized by Eurofins Genomics K.K. (Tokyo, Japan)). After restriction enzyme digestion, the coding fragment was incorporated into the pET-15b vector digested with *Nde*I and *Bam*HI (Novagen, Madison, WI), where the first methionine of *A. pernix* Trbp was fused into the *Nde*I site. The DNA sequence was confirmed by Eurofins Genomics K.K. (Tokyo, Japan). 

### 2.2. Expression and Purification of A. pernix Trbp

*Escherichia coli* BL21-Codon Plus(DE3)-RIL (Stratagene, La Jolla, CA, USA) transformed with *A. pernix* Trbp expression plasmid was grown to confluence and induced with 0.5 mM isopropyl-β-D-thiogalactoside (IPTG). Cultures were harvested and cells were suspended in lysis buffer (20 mM Tris-HCl (pH 7.4), 300 mM NaCl, 10 mM imidazole), followed by the disruption of the cells by sonication on ice. The supernatant by centrifugation was collected and heated to 90 °C for 1 h and re-centrifuged. The resulting supernatant was loaded onto a Ni-NTA agarose column (QIAGEN, Valencia, CA, USA). After washing with wash buffer (20 mM Tris-HCl (pH 7.4), 300 mM NaCl, 20 mM imidazole), the proteins were eluted with elution buffer (20 mM Tris-HCl (pH 7.4), 300 mM NaCl, 250 mM imidazole). The sample was applied on Blue Sepharose 6 Fast Flow (GE Healthcare, Uppsala, Sweden) column equilibrated with BS lysis buffer (20 mM Tris-HCl (pH 7.4), 20 mM NaCl). After washing with BS wash buffer (20 mM Tris-HCl (pH 7.4), 300 mM NaCl), the proteins were eluted with BS elution buffer (20 mM Tris-HCl (pH 7.4), 1 M NaCl). Fractions containing a homogeneous protein enzyme were pooled and dialyzed twice in 1 L of dialysis buffer (20 mM Tris-HCl (pH 7.4), 100 mM NaCl) followed by concentration by Centriprep Ultracel YM-10 (Millipore Ireland B.V., Carrigtwohill, Ireland). Finally, the proteins were stored in 50% glycerol.

### 2.3. Preparation of RNA Pools for in Vitro Selection of Aptamers

Template DNAs for RNA pools and primers for PCR were obtained from Eurofins Genomics K.K. (Tokyo, Japan). The template N30H DNA (5′-GGTAGATACGATGGA-N30-CATGACGCGCAGCCA-3′; constant region for the PCR and random sequences are shown) was amplified using N30H-forward primer (5′-TGTAATACGACTCACTATAGGTAGATACGATGGA-3′; T7 promoter sequences are underlined) and N30H-reverse primer (5′-TGGCTGCGCGTCATG-3′) [14]. RNA transcription was performed at 42 °C for 3 h in a reaction mixture containing 40 mM Tris-HCl (pH 8.0), 10 mM dithiothreitol, 2 mM spermidine, 8 mM MgCl_2_, 2.5 mM each NTP, template DNA (0.2 mg/mL), and pure T7 RNA polymerase (~100 μg/mL) [15,16]. The transcripts were recovered by ethanol precipitation and were treated with DNase I (epicentre, Madison, WT, USA). Then, the transcripts were purified by denaturing 12% polyacrylamide gel electrophoresis. The concentrations of the obtained purified RNA were determined from the ultraviolet absorbance at a wavelength of 260 nm using Implen NanoPhotometer (München, Germany).

### 2.4. A. pernix Trbp Binding Assay Using Nitrocellulose Filter

*A. pernix* Trbp was bound to a nitrocellulose filter (0.45 μm, Millipore, Bedford, MA, USA), and selection was carried out for specific *A. pernix* Trbp and RNA pools shown in Table 1. RNA solution (40 μL) was heat-denatured at 95 °C for 2 min, cooled to room temperature for 15 min, and 10 μL of 5 × binding buffer (225 mM Tris-HCl (pH 7.5), 25 mM MgCl_2_, and 1 M NaCl) were added. *A. pernix* Trbp (Table 1) was bound to the nitrocellulose filter and pre-equilibrated with binding buffer (25 mM Tris-HCl (pH 7.5), 5 mM MgCl_2_, and 200 mM NaCl). The filter was washed three times with 50 μL binding buffer, and incubated in RNA solution at room temperature for 30 min. The filter was then washed with fresh binding buffer (Table 1). The washed filter was placed in a 1.5 mL microcentrifuge tube, and 7 M urea was then added. The filter was heated at 95 °C for 5 min to dissociate the RNA bound to *A. pernix* Trbp. The dissociated RNAs were recovered by ethanol precipitation and reverse transcribed using ReverTra Ace (TOYOBO, Osaka, Japan) at 42 °C for 1 h. The dsDNA product was amplified by PCR, transcribed *in vitro* by T7 RNA polymerase, and purified on 12% polyacrylamide gel electrophoresis (PAGE) containing 7 M urea. Purified RNAs were used for the next cycle of the selection.

After 13 rounds of PCR, the amplicon was cloned into the pTAC-1 vector (BioDynamics Laboratory Inc., Tokyo, Japan) and transformed into *E. coli* DH5α. Plasmid DNA was isolated from individual clones and sequenced by Eurofins Genomics K.K. (Tokyo, Japan). The secondary structure models of the selected aptamers were drawn using the CentroidFold web server (provided by CBRC, AIST, Japan) (http://rtools.cbrc.jp/centroidfold) [17,18]. CONTRAfold was used as the inference engine, and 2^2^ was used as the weight of base pairs.

### 2.5. Binding Assessments of RNA Pools Using PCR

*A. pernix* Trbp binding assay was performed as described above using 1 pmol of RNA prior to PCR (G0) and after the 6th (G6) and 13th (G13) cycles. Nitrocellulose filters were washed with 5 mL binding buffer. The purified, dissociated RNAs were reverse transcribed, and the dsDNA product was amplified by PCR, and an aliquot of every third cycle (up to 15 cycles total) was analyzed by 2% agarose gel electrophoresis with 0.5 μg/mL ethidium bromide. As a negative control, *A. pernix* Trbp-free nitrocellulose filter was incubated in RNA solution in the G13 pool.

### 2.6. Electrophoretic Mobility Shift Assay (EMSA)

A volume of 10–30 μM of each RNA (aptamers and their deletion mutants) and 10 μM *A. pernix* Trbp were mixed in binding buffer and incubated at 4 °C for 30 min. (Here, 100 pmol of *A. pernix* Trbp was added to 10 μL of the solution.) After the addition of an equal volume of 40% glycerol, the solution was analyzed by electrophoresis on non-denaturing 8% polyacrylamide gels containing 44.5 mM Tris, 44.5 mM borate, and 10 mM MgCl_2_ [19]. The gel was stained with 0.04% toluidine blue. Destaining conditions and color tone correction were dependent on individual gels.

### 2.7. Selective 2’-Hydroxyl Acylation Analyzed by Primer Extension (SHAPE)

The procedure is based on the method reported by Weeks and coworkers with slight modifications [20]. RNA (50 μM) in 3 μL of sterile water was heated at 95 °C for 3 min, quickly cooled on ice, treated with 1.5 μL of folding buffer (333 mM NaCl, 333 mM HEPES (pH 8.0), 33.3 mM MgCl_2_), and incubated at 37 °C for 20 min. The RNA solution was treated with N-methylisatoic anhydride (NMIA) (0.5 μL, 130 mM in anhydrous DMSO, 37°C), allowed to react for 60 min, and placed on ice. Modified RNAs were subjected to primer extension without further purification. Modified RNA (2 μL) was added to 8 μL of 10 μM 5ʹ-6-carboxyfluorescein (FAM)-labeled reverse transcription DNA primer (5ʹ-FAM-GAACCGGACCsGAAGCCCG-3ʹ) prepared by Japan Bio Services Co., Ltd. (Saitama, Japan), and heated to 65 °C (6 min), and incubated at 37 °C (20 min). A volume of 4 μL of 5 × ReverTra Ace buffer, 4 μL of 2.5 mM each dNTP, and 0.25 μL of water were added. The RNA solution was heated to 42 °C. 0.25 μL of ReverTra Ace was added and mixed by gentle pipetting, and reactions were incubated at 42 °C for 60 min. Primer extension reactions were quenched by addition of 1 μL of 4 M NaOH, heating (65 °C for 10 min), subsequent addition of 2 μL of 2 M HCl for neutralizing [11]. The solution was applied to denaturing 16% polyacrylamide gel for electrophoresis. The gel was analyzed on a Typhoon FLA 7000 (GE Healthcare Japan, Tokyo, Japan) [21].

## 3. Results

### 3.1. Selection of A. pernix Trbp Aptamers

**Figure 2:**
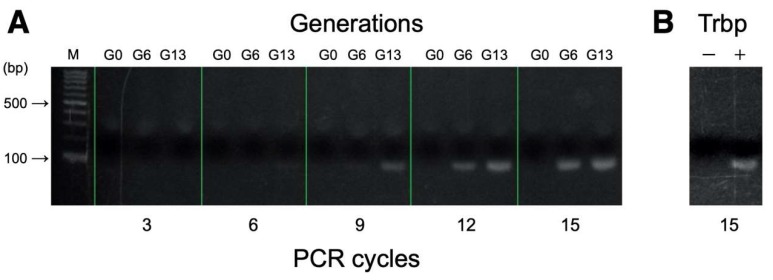
(**A**) The number of PCR cycles needed to detect the amplified DNA from *A. pernix* Trbp aptamer-dependent reverse transcribed DNA between generations (G0, G6, and G13). (**B**) Amplified DNA detection after 15 PCR cycles in the absence (−) and presence (+) of *A. pernix* Trbp.

RNA aptamers specific for *A. pernix* Trbp were successfully selected from a pool of 60-nucleotide RNAs that consist of a 30-nucleotide random region. The binding process was evaluated by comparing the number of PCR cycles needed to detect the amplified reverse-transcribed DNA from *A. pernix* Trbp aptamers between subsequent generations (Figure 2A). No band was detected for generation 0 (G0) even after 15 cycles of PCR. Earliest band detection was found at G6 after 12 PCR cycles. At G13, the expected band was detected after 9 PCR cycles. In addition, no DNA amplification was observed in the absence of Trbp even after 15 PCR cycles, suggesting that the aptamers were specific to Trbp (Figure 2B).

**Figure 3:**
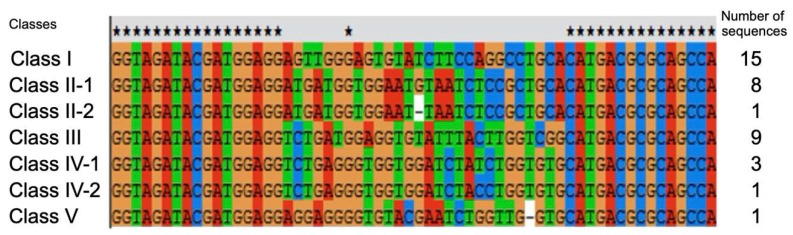
RNA aptamer sequences derived from the 13 rounds of *A. pernix* Trbp-binding selection. Black star (★) indicates conserved sequences including the flanking 5′-end and 3′-end. Left: classifications derived from the obtained sequences. Right: number of sequences per class.

**Figure 4:**
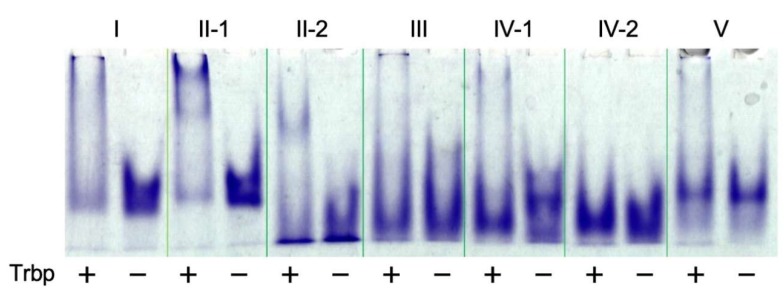
Gel shift assay of each class of RNA aptamers with (+; 10 μM) and without (−) *A. pernix* Trbp.

Sequencing results following 13 rounds of *A. pernix* Trbp-binding selection are shown in Figure 3. Among 38 individual sequences, many were found multiple times, and were categorized as Class I–V. We also identified three unique sequences that were classified as individual classes. The aptamers derived from the representative sequence of each class were then verified for Trbp-binding ability (Figure 4). In the absence of *A. pernix* Trbp, the position of the bands of Class I, Class II-1, and Class V differed from that of other classes, suggesting dimer formation among these aptamers. 

Class III and Class IV-1 also seemed to form a dimer independently of *A. pernix* Trbp. In the presence of 100 pmol of *A. pernix* Trbp, band shifts were detected in Class I and Class II-1 and relatively deeper bands were observed. Faint band shifts were also detected in Class III. Class II-2 had a high monomer ratio, and band shifts were observed with high *A. pernix* Trbp concentration, although the shift was of a lower magnitude than that of other classes. Most of Class IV-2 existed as monomers, and no large band shift was detected even in the presence of *A. pernix* Trbp.

### 3.2. Reduction of Class II-1 and Class II-2 Aptamers Based on Their Secondary Structure

**Figure 5:**
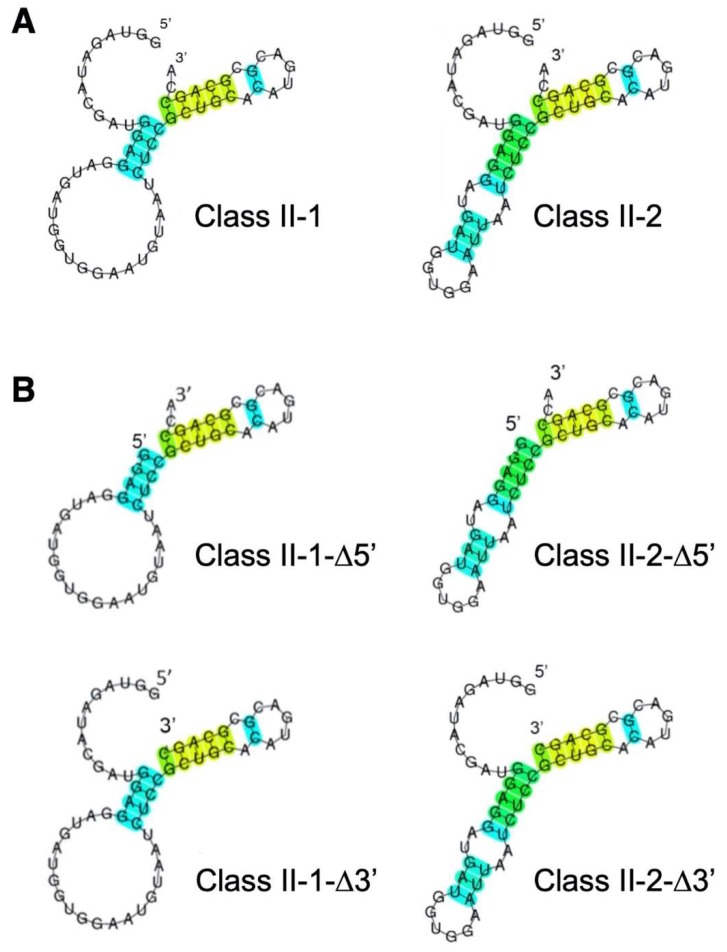
Secondary structure prediction of the obtained RNA aptamers using CentroidFold. (**A**) Class II-1 and Class II-2; (**B**) Deletion mutants of the single stranded region of 5′- or 3′-end of Class II-1 and Class II-2 (Class II-1-Δ5′, Class II-2-Δ5′, Class II-1-Δ3′, and Class II-2-Δ3′). Blue-to-red heat color gradation is used to demonstrate probability of the predicted base pairs from 0 to 1.

Because Class II-1 had the strongest binding (Figure 4), and Class II-1 and II-2 only differ on the presence of the G31 nucleotide, we focused on these two aptamers hereafter. The secondary structures of Class II-1 and Class II-2 were predicted using CentroidFold (Figure 5A). Both have similar stem and loop structures with single stranded 5′-twelve-nucleotide and 3′-dinucleotide (CA) (Figure 5A). Their secondary structures differ in the size of their central loop.

**Figure 6:**
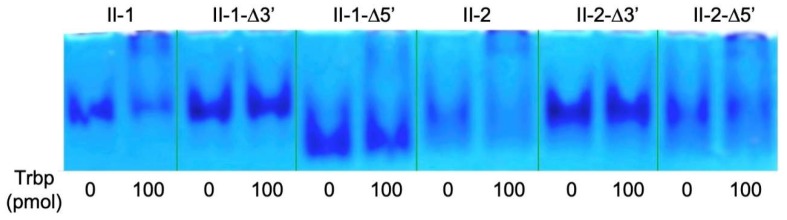
Gel shift assay of the aptamers in Class II and their mutants in the absence and the presence (100 pmol) of *A. pernix* Trbp.

**Figure 7:**
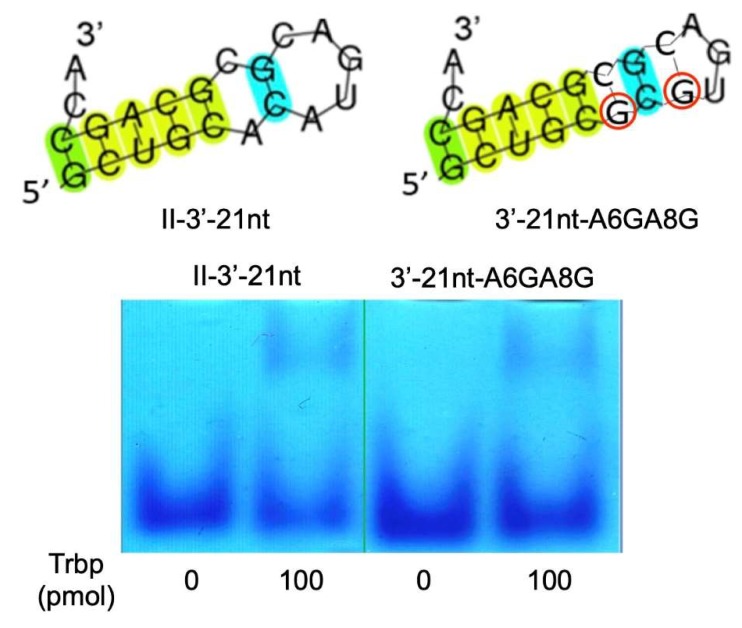
Secondary structure prediction of Class II-3′-21nt and its mutant (Class II-3′-21nt-A6GA8G) and gel shift assay of these aptamers in the absence and the presence (100 pmol) of *A. pernix* Trbp.

Based on the secondary structures, we prepared deletion mutants of the single-stranded 5′- or 3′-regions (Figure 5B). Class II-1-Δ5′ and Class II-2-Δ5′ mutants, both of which are deficient for the 12-nucleotide region at the 5′-end, were bound to *A. pernix* Trbp (Figure 6); however, Class II-1-Δ3′ and Class II-2-Δ3′ mutants lacking 2-nucleotide at the 3′-end failed to bind to *A. pernix* Trbp (Figure 6). This suggests that the 3′-terminal CA nucleotides are important for binding to *A. pernix* Trbp. Next, we focused on the secondary structures of Class II-1-Δ5′ and Class II-2-Δ5′. The last 21 nucleotides common to both Class II-1-Δ5′ and Class II-2-Δ5′ were isolated (Figure 7). The resulting RNA molecule (Class II-3′-21nt) was able to bind to *A. pernix* Trbp (Figure 7).

To identify the Trbp-binding region of Class II-3′-21nt, further mutations were prepared. Class II-3′-21nt-A6GA8G is a mutant of Class II-3′-21nt in which both A6 and A8 nucleotides were replaced with G nucleotides. These point mutations are expected to close the Class II-3′-21nt loop (Figure 7). However, mutations at this loop did not affect the binding to *A. pernix* Trbp (Figure 7). 

**Figure 8:**
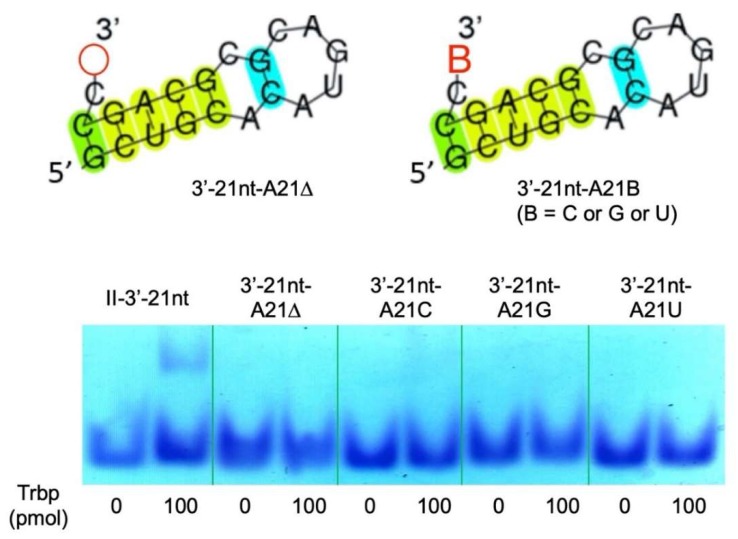
Deletion or substitution mutants of the 3′-terminal A of Class II-3′-21nt (Class II-3′-21nt-A21Δ, Class II-3′-21nt-A21C, Class II-3′-21nt-A21G, and Class II-3′-21nt-A21U), and gel shift assay of these aptamers in the absence and the presence (100 pmol) of *A. pernix* Trbp.

Next, we focused on the 3′-terminal CA nucleotides of Class II-3′-21nt and prepared deletion and substitution mutants of the 3′-terminal A nucleotide (A21), resulting in Class II-3′-21nt-A21Δ, Class II-3′-21nt-A21C, Class II-3′-21nt-A21G, and Class II-3′-21nt-A21U mutant RNA molecules (Figure 8). Deletion or substitution of the 3′-terminal A inhibited binding to *A. pernix* Trbp, although Class II-3′-21nt-A21C still yielded a very faint band (Figure 8). These results suggest that the 3′-terminal A is important for binding to *A. pernix* Trbp.

**Figure 9:**
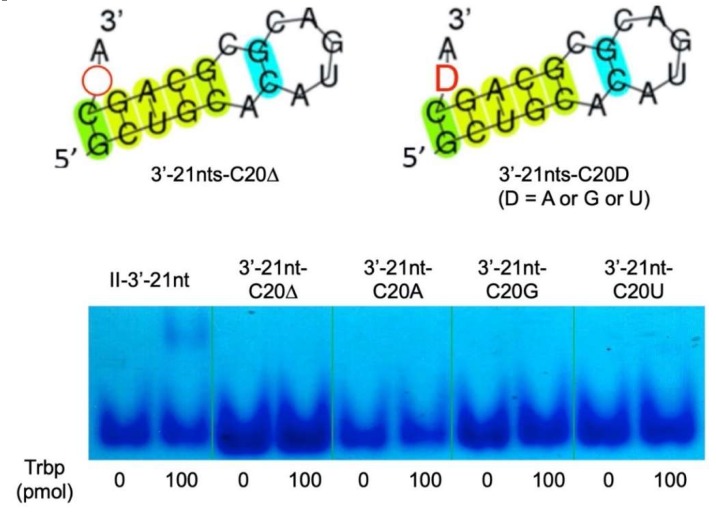
Deletion or substitution mutants of C20 of Class II-3′-21nt (Class II-3′-21nt-C20Δ, Class II-3′-21nt-C20A, Class II-3′-21nt-C20G, and Class II-3′-21nt-C20U) and gel shift assay of these aptamers in the absence and the presence (100 pmol) of *A. pernix* Trbp.

We also prepared deletion and substitution mutants of the C20 nucleotide of Class II-3′-21nt, resulting in Class II-3′-21nt-C20Δ, Class II-3′-21nt-C20A, Class II-3′-21nt-C20G, and Class II-3′-21nt-C20U mutant RNA molecules (Figure 9). *A. pernix* Trbp did not bind any of these mutants (Figure 9), suggesting that the CA nucleotides at the 3′-end are both important for binding to *A. pernix* Trbp.

### 3.3. Secondary Structure of Class II-3′-21nt Analyzed by SHAPE 

**Figure 10:**
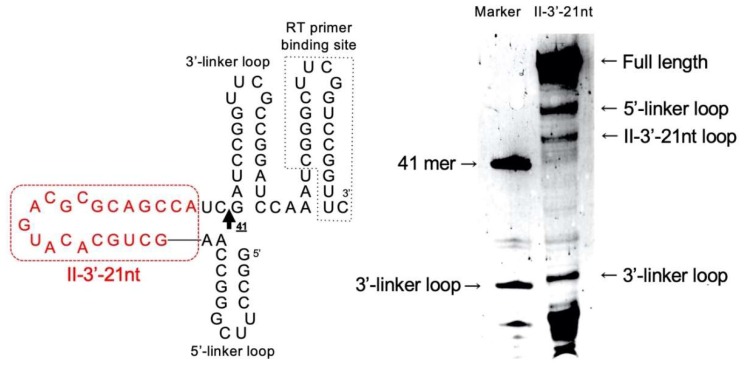
Left: Schematic presentation of Class II-3′-21nt in the context of an RNA structure cassette for SHAPE experiment. The 5′-and 3′-linker regions facilitate analysis of sites of 2′-O-adduct formation by primer extension and fold, independently of Class II-3′-21nt. Right: SHAPE analysis of Class II-3′-21nt embedded within a structure cassette. As a marker, reverse transcriptase-mediated primer extension product (without NMIA) based on the transcript of 41-mer was used.

The computational prediction of the secondary structure of Class II-3′-21nt shown in Figure 7 may not reflect tertiary structures. To clarify the structure, we performed SHAPE analysis (Figure 10). SHAPE is used for probing RNA secondary structures. Nucleophilic reactivity of 2′-hydroxyl groups in flexible regions is enhanced toward electrophiles, such as NMIA. At the modified sites, primer extension reaction halts, and the resulting fragments are detected by electrophoretic separation [20]. As shown in Figure 10, bands corresponding to the 3′-linker loop, Class II-3′-21nt loop, 5′-linker loop, and full-length (78-mer) molecules were confirmed. The expected structure of Class II-3′-21nt is relatively simple, and the SHAPE signals derived between the bands detected for the 5′-linker loop and the 41-mer marker support the computational prediction of the secondary structure of Class II-3′-21nt.

## 4. Discussion

EMSA is a rapid and sensitive method to detect protein-nucleic acid interactions for qualitative applications. In this study, we identified structural features of RNA molecules that bind to *A. pernix* Trbp prior to quantitative analysis. The result of the native PAGE assay suggests that Class II-1 forms a dimer, whereas Class II-2 binds as a monomer. Dimer formation is not essential for binding to *A. pernix* Trbp; instead, the 3′-terminal single stranded CA nucleotides are the most important binding determinant. The N30H pool has been successfully used and fixed primer sequences were previously determined for general aptamer isolation applications [14]. Therefore, we did not specifically design the primer binding sequence. In addition, although the third nucleotide from the 3′-end (C) normally pairs with the 5′-end G nucleotide on the opposite strand, it may also remain single stranded along with the adjoining CA nucleotides, forming a structure similar to modern tRNA molecules. The thermodynamic contribution of the G-C base pair at the end of a helix is estimated to be roughly -5 kcal/mol [22]. Therefore, both conformations can stably exist at room temperature.

*A. aeolicus* Trbp111-like structures are frequently found aaRSs and a broad range of protein types across different organisms [1,5,6,7,8]. Multiple alignments of *A. pernix* Trbp with *A. aeolicus* Trbp111 and *N. equitans* MetRS-C (Figure 1) indicate that these three proteins likely possess similar tertiary structures. Accordingly, we previously solved the structure of *N. equitans* MetRS-C and found that it was similar to *A. aeolicus* Trbp111, both of which have an OB-fold [11], one of the most evolutionarily ancestral folds inferred from the phylogenomic analysis of protein architecture [23].

*A. aeolicus* Trbp111 has been suggested to bind to the corner of L-shaped tRNA, consistent with an evolutionary role in contributing to the formation of full-length tRNA through the binding of the two segments [3,4,24]. However, from the binding property of half-sized tRNA [11], we found that *N. equitans* MetRS-C preferably binds to the split 3′-half tRNA species than that of the 5′-half species [11]. These results suggest that the structure for binding to MetRS-C might be a minihelix-like stem-loop with a single-stranded terminus.

A ubiquitous protein such as *A. aeolicus* Trbp111 and *A. pernix* Trbp might have played an evolutionary role through the interactions of key RNA structures and proteins. In the present *in vitro* selection study, the structure for binding to *A. pernix* Trbp was a hairpin stem-loop with a single-stranded 3′-terminal, consistent with the results from our previous study of *N. equitans* MetRS-C [11]. More importantly, both nucleotides of the 3′-terminal CA were necessary for the binding to *A. pernix* Trbp, which may be related to the evolutionarily conserved role of the CCA-3′ terminus of tRNA in mediating the interaction with Trbp-like structures. Therefore, *A. pernix* Trbp can bind one arm of the L-shaped region of tRNA, including the CCA-3′ terminus, instead of the corner region of this structure. Hairpin RNAs with CCA-3′ termini have also been suggested to be related to the origin of homochiral aminoacylation in the RNA world [25,26,27,28]. During evolution, the full-length L-shaped structure of tRNA might have been formed from half-sized RNAs [29,30,31,32].

Quantitative features of the binding remain unsolved because of limitations of the methods used in this study. The faint band found in EMSA analysis of Class II-3′-21nt may be due to weak binding to *A. pernix* Trbp or to weak staining of the shortened construct. Quantitative and mutational analysis of *A. pernix* Trbp with respect to RNA binding will be required in the future.

## Figures and Tables

**Table 1:** Amount of *A. pernix* Trbp and RNA used in each selection cycle, and the volume and number of washes per cycle.

Generation	*A. pernix* Trbp (pmol)	RNA (pmol)	Wash (mL × times)
1	40	1000	0.5 × 3
2	10	100	0.5 × 3
3	1	10	0.5 × 3
4	1	10	0.5 × 3
5	0.5	5	0.5 × 3
6	0.3	3	0.5 × 3
7	0.3	3	0.5 × 3
8	1	1	5 × 1
9	1	1	5 × 1
10	1	1	5 × 1
11	1	1	5 × 1
12	1	1	5 × 1
13	1	1	5 × 1
